# Sichtweisen einer Stichprobe überwiegend jüngerer Ärztinnen und Ärzte zum ärztlich-assistierten Suizid

**DOI:** 10.1007/s00103-024-03833-5

**Published:** 2024-01-22

**Authors:** Remo Küppers, Stefan Meier, Yann-Nicolas Batzler, Manuela Schallenburger, Dietmar Wetzchewald, Sven Dreyer, Jaqueline Schwartz, Martin Neukirchen

**Affiliations:** 1 Klinik für Anästhesiologie, Heinrich-Heine-Universität Düsseldorf, Universitätsklinikum Düsseldorf, Moorenstr. 5, 40225 Düsseldorf, Deutschland; 2 Interdisziplinäres Zentrum für Palliativmedizin, Heinrich-Heine-Universität Düsseldorf, Universitätsklinikum Düsseldorf, Düsseldorf, Deutschland; 3 Centrum für Integrierte Onkologie Aachen Bonn Cologne Düsseldorf (CIO ABCD), Düsseldorf, Deutschland; 4 Institut für Notfallmedizin, Universität Witten/Herdecke, Arnsberg, Deutschland; 5 Klinik für Orthopädie und Unfallchirurgie, Heinrich-Heine-Universität Düsseldorf, Universitätsklinikum Düsseldorf, Düsseldorf, Deutschland

**Keywords:** Suizidalternativen, Suizidassistenz, § 217 StGB, FVET, Sterbehilfe, Suicide alternatives, Suicide assistance, § 217 StGB, FVET, Euthanasia

## Abstract

**Hintergrund:**

Am 26.02.2020 hat das Bundesverfassungsgericht den § 217 StGB, der die geschäftsmäßige Förderung der Selbsttötung unter Strafe stellte, für nichtig erklärt. Seitdem wird eine mögliche gesetzliche Neuregelung diskutiert. Ziel dieser Studie war es, Wissen, Erfahrungen und Einstellungen jüngerer Ärztinnen und Ärzte zur Suizidassistenz zu untersuchen.

**Methoden:**

Von November 2022 bis März 2023 wurde eine quantitative Umfrage in Fortbildungskursen zur Notfallmedizin, Intensivmedizin und Hämatologie durchgeführt und deskriptiv ausgewertet.

**Ergebnisse:**

Insgesamt wurden 1163 Datensätze (Rücklaufquote 82,1 %) ausgewertet. 90,8 % der Befragten hatten bereits Sterbende betreut. 62,3 % befürworteten eine Suizidassistenz nur bei Menschen in palliativen Behandlungssituationen, 20,1 % befürworteten Suizidassistenz unabhängig vom Gesundheitszustand des Menschen. 33,1 % wurden bereits um Suizidassistenz gebeten. 3,3 % hatten persönlich bei einer Suizidassistenz mitgewirkt. 71,0 % kannten den Inhalt des Bundesverfassungsgerichtsurteils zum § 217 nicht, 72,0 % waren über die Gesetzesvorschläge zur Neuregelung des assistierten Suizids nicht informiert. 66,4 % sahen Ärztinnen und Ärzte als die richtigen Ansprechpartner, um über die Zulässigkeit eines Suizidwunsches zu entscheiden.

**Diskussion:**

Die Untersuchung zeigt, dass jüngere Ärztinnen und Ärzte einen deutlichen Unterschied in ihrer Haltung zur Suizidassistenz zwischen Menschen ohne Vorerkrankungen und solchen in palliativen Behandlungssituationen machen. Weitere Untersuchungen über die Ursachen der häufigen Unkenntnis der normativen Grundlagen sind notwendig. Die Ergebnisse legen nahe, dass mehr Aufklärungsarbeit über Suizidalternativen und palliativmedizinische Versorgungsmöglichkeiten geleistet werden muss.

## Abstract (en)

**Background:**

On 26 February 2020, the German Federal Constitutional Court revoked a law (§ 217 StGB) that had banned assisted suicide intended to be repeated on a regularly basis. Since then, a possible new legal regulation has been discussed. This study examined the knowledge, experiences, and attitudes of younger physicians towards physician-assisted suicide (PAS).

**Methods:**

A quantitative survey of postgraduate courses in emergency medicine, critical care medicine, and hematology was conducted from November 2022 to March 2023. Possible factors influencing attitudes towards PAS were analyzed.

**Results:**

A total of 1163 records (response rate 82.1%) were analyzed. Of the participants, 90.8% had experience with dying patients, 62.3% supported PAS only in palliative scenarios, 20.1% supported PAS regardless of the health status, 33.1% have been asked for PAS, and 3.3% had participated in PAS. In addition, 71.0% did not know the content of the Federal Constitutional Court’s decision on § 217, 72.0% were not informed about the legislative proposals for the new regulation of PAS, and 66.4% saw doctors as the right person to decide whether a suicidal wish is permissible.

**Conclusion:**

Younger doctors differentiate in their attitude towards PAS between people without illnesses and those in palliative treatment situations. Further investigations into the causes of the frequent ignorance of the normative foundations are necessary. The results suggest that more educational work needs to be done about suicide alternatives and palliative care options.

## Einleitung

Am 26.02.2020 hat das *Bundesverfassungsgericht* (BVerfG) den § 217 StGB, der die geschäftsmäßige Förderung der Selbsttötung unter Strafe stellte, für nichtig erklärt, da diese Regelung zu weit ins allgemeine Persönlichkeitsrecht eingreife [1]. Die gesetzliche Regelung ist nun dieselbe wie vor 2015, als der § 217 StGB verabschiedet wurde. Suizidassistenz ist damit prinzipiell legal, in vielen Details jedoch unreguliert. Es gibt eine politische, gesellschaftliche und innerärztliche Debatte über eine mögliche gesetzliche Neuregelung der Suizidassistenz. Vom Parlament wurden ursprünglich 3 fraktionsübergreifende Gesetzentwürfe vorgelegt, von denen 2 eher liberale Anträge zusammengelegt wurden [1–4]. Keiner dieser Anträge fand in der Bundestagsabstimmung vom 06.07.2023 eine erforderliche Mehrheit.

Die Debatte über die Neuregelung des § 217 wird sowohl parlamentarisch [5] als auch in Teilen der Gesellschaft und insbesondere innerärztlich kontrovers geführt. So hält die *Deutsche Gesellschaft für Palliativmedizin* (DGP) die aktuelle Rechtslage für ausreichend. Vordringlich sei eine vermehrte Aufklärung der Bevölkerung über die suizidpräventiv wirkenden Möglichkeiten einer qualitativ hochwertigen palliativmedizinischen Versorgung [6]. Hierbei sollte unter anderem auch über Alternativen wie den „freiwilligen Verzicht auf Essen und Trinken“ (FVET) informiert werden. Die *Deutsche Gesellschaft für Psychiatrie und Psychotherapie, Psychosomatik und Nervenheilkunde* (DGPPPN) fordert hingegen, dass über die Zulässigkeit einer Suizidassistenz im konkreten Fall Gerichte und nicht etwa Ärztinnen und Ärzte zu entscheiden hätten [7]. Unter anderem die *Bundesärztekammer* (BÄK) führt an, dass eine Erlaubnis des ärztlich assistierten Suizids das Vertrauen in die Ärzteschaft schädige [8]. Viele Wortmeldungen stimmen darin überein, dass Suizidassistenz grundsätzlich keine ärztliche Aufgabe sei [6–8]. Der 127. Deutsche Ärztetag hat die Prüfung folgender Ergänzung der Musterberufsordnung beschlossen: „Die Mitwirkung bei der Selbsttötung (assistierter Suizid) ist grundsätzlich keine ärztliche Aufgabe. Sie ist bei schwerer oder unerträglicher Erkrankung nach wohlabgewogener Gewissensentscheidung im Einzelfall zulässig.“ Jede Beratung von Sterbewilligen, ohne dass Krankheit die Grundlage des Sterbewillens darstellte, würde so außerhalb eines Arzt-Patienten-Verhältnisses stattfinden [9].

Die Einstellungen und Haltungen jüngerer Ärztinnen und Ärzte zum assistierten Suizid sind bislang nur wenig bekannt. Aus verschiedenen Erhebungen geht hervor, dass Medizinstudentinnen und -studenten lebensbegrenzenden Maßnahmen tendenziell häufiger zustimmen als approbierte Ärztinnen und Ärzte [10, 11]. Klinikärztinnen und -ärzte, die im Schnitt jünger an Lebens- und Berufsjahren sind, vertreten häufiger eine zur Suizidassistenz zustimmende Einstellung als die meist älteren niedergelassenen Ärztinnen und Ärzte [12–14]. Interessanterweise nimmt die Zustimmungsrate zur Suizidassistenz im höheren Lebensalter wieder zu [14].

An verschiedenen Kollektiven wurden Einflussfaktoren auf die Einstellung zum assistierten Suizid untersucht, u. a. die Religionszugehörigkeit und Religiosität, das Lebensalter, die Erfahrung mit Sterbenden, die Jahre der Berufstätigkeit sowie die Fachrichtung [15–19]. Es ist nicht bekannt, wie stark diese Einflussfaktoren mit den Haltungen jüngerer Ärztinnen und Ärzte korrelieren.

In der vorliegenden Studie wurden Haltungen junger Ärztinnen und Ärzte zum assistierten Suizid erfragt. Die Daten wurden auf mögliche Einflussfaktoren wie Vorerfahrungen, Vorkenntnisse oder demografische Merkmale untersucht.

## Methoden

Für die vorliegende Befragung wurde von unserer Arbeitsgruppe auf Basis einer Literaturrecherche ein Fragenkatalog entwickelt, der ausgewählte Aspekte um die Debatte zur Suizidassistenz abbildet (siehe Onlinematerial 1). Die Fragen wurden in einer interdisziplinären Expertengruppe abgestimmt und auf Basis von Prätests auf Inhalt und Verständlichkeit geprüft. Die Fragen wurden mittels *SoSci Survey* (SoSci Survey GmbH, München, Deutschland) zu einem Onlinefragebogen zusammengestellt.

Die demografischen Daten wurden über Multiple-Choice- bzw. Multiple-Select-Fragen erhoben. Die Antworten zu den restlichen Fragen wurden mithilfe einer 5‑stufigen Likert-Skala (ordinal) erhoben, die sowohl zustimmende („trifft voll zu“ + „trifft eher zu“ bzw. „stimme voll zu“ bzw. „stimme eher zu“) als auch ablehnende Antworten („trifft gar nicht zu“ + „trifft eher nicht zu“ bzw. „stimme gar nicht zu“ bzw. „stimme eher nicht zu“) ermöglichte.

### Studienpopulation und Datenanalyse

Die Studienpopulation wurde aus den Teilnehmerinnen und Teilnehmern von Einführungskursen zum Thema Intensivmedizin, Weiterbildungskursen zum Erwerb der Zusatzbezeichnung Notfallmedizin sowie dem Kurs „Hämatologie kompakt“ der Arbeitsgemeinschaft Intensivmedizin e. V. in Arnsberg rekrutiert. Eingeschlossen wurden von November 2022 bis März 2023 alle Teilnehmerinnen und Teilnehmer der in Tab. 1 genannten Präsenzkurse. Sie wurden von dem Dozenten über die Studie informiert. Der Link zur Onlineumfrage wurde ihnen im Rahmen ihrer Fortbildung zugänglich gemacht. Teilnehmerinnen und Teilnehmer, die den Fragebogen nicht vollständig bearbeiteten, wurden ausgeschlossen. Nach systematischer Aufbereitung wurden die Daten mit Microsoft Excel 365, Version 2304 (Microsoft Inc., Redmond, WA, USA), und SPSS Statistics 29 (IBM Inc., Armonk, NY, USA) deskriptiv analysiert. Anschließend erfolgten explorative Analysen möglicher Einflussfaktoren auf die Haltungen zur Suizidassistenz. Korrelationsmaße wurden mittels Spearman (ordinal vs. ordinal) oder Cramers V (nominal vs. ordinal) ermittelt.

**Table 1:** 

	„Seminarkongress Notfallmedizin“	„Einführungskurs Intensivmedizin“	„Hämatologie kompakt“
Kursinhalt und -ziel	Inhalte entsprechend den Ausbildungsrichtlinien der BÄK zur Erlangung der ZB Notfallmedizin	Vermittlung von Grundlagenwissen der allgemeinen und speziellen Intensivmedizin	Kompaktwissen hämatologische Erkrankungen
Teilnahmebedingung laut Veranstalter AIM	Ärztinnen und Ärzte mit ≥ 1 Jahr Berufserfahrung	Ärztinnen und Ärzte am Beginn ihrer Intensivzeit	Hämatologisch interessierte Ärztinnen und Ärzte jeglichen Ausbildungsstandes
Teilnehmerzahl (kumulativ)	634 (verteilt auf 3 Kurse)	723 (verteilt auf 3 Kurse)	60 (1 Kurs)

*BÄK* Bundesärztekammer, *ZB* Zusatzbezeichnung, *AIM* Arbeitsgemeinschaft Intensivmedizin e. V.

**Table 2:** 

	Gesamt *n* [%]	Notfallmedizin *n* [%]	Intensivmedizin *n* [%]	Hämatologie *n* [%]	Bevölkerung BRD [%]
*Geschlecht*^a^					BRD 2022
Weiblich	612 [53,4]	228 [46,2]	353 [58,4]	31 [63,3]	[50,7]
Männlich	534 [46,6]	265 [53,6]	251 [41,6]	18 [36,7]	[49,2]
Divers	1 [0,1]	1 [0,2]	0	0	[< 0,1]
Keine Angabe	16 [1,4]	–	–	–	–
*Klinisch tätig seit *^a^					
0–2 Jahren	433 [37,2]	165 [33,1]	263 [42,8]	5 [10,0]	–
3–5 Jahren	592 [50,9]	264 [52,9]	315 [51,3]	13 [26,0]	
6–10 Jahren	95 [8,2]	45 [9,0]	22 [3,6]	28 [56,0]	
11–20 Jahren	25 [2,1]	14 [2,8]	8 [1,3]	3 [6,0]	
> 20 Jahren	18 [1,5]	11 [2,2]	6 [1,0]	1 [2,0]	
*Religion*					BRD 2021
Konfessionsfrei	348 [32,4]	147 [31,7]	183 [32,3]	18 [40,0]	[42]
Christlich	577 [53,7]	248 [53,4]	305 [53,9]	24 [53,3]	[52]
Muslimisch	127 [11,9]	60 [12,9]	65 [11,5]	2 [4,4]	[3,5–6,7] ^d^
Buddhistisch	7 [0,7]	4 [0,9]	3 [0,5]	0	[0,3]
Jüdisch	3 [0,3]	1 [0,2]	2 [0,4]	0	[0,2]
Andere	13 [1,2]	4 [0,9]	8 [1,4]	1 [2,2]	[0,5]
Keine Angabe	88 [7,6]	–	–	–	–
Gesamt	1163 [100]	499 [42,9]	614 [52,8]	50 [4,3]	–
*Fachrichtung *^a,c^					
Innere Medizin	667 [56,4]	222 [44,5]	397 [64,7]	48 [96,0]	–
Anästhesiologie	310 [26,2]	173 [34,7]	137 [22,3]	0	
Chirurgie	102 [8,6]	58 [11,6]	42 [6,8]	0	
Neurologie	57 [4,8]	23 [4,6]	34 [5,5]	0	
Andere	46 [3,9]	31 [6,2]	10 [1,6]	5 [10,0]	
*Facharztstatus *^a^					
Ja	78 [6,7]	39 [7,8]	15 [2,4]	24 [48,0]	–
Nein	1085 [93,3]	460 [92,2]	599 [97,6]	26 [52,0]	
*Zusatzbezeichnung *^b,c^					
ZB Notfallmedizin	83 [7,0]	31 [6,2]	41 [6,7]	9 [18,0]	–
ZB Intensivmedizin	32 [2,7]	10 [2,0]	22 [3,6]	0	
ZB Palliativmedizin	21 [1,8]	3 [0,6]	10 [1,6]	8 [16,0]	
ZB Geriatrie	4 [0,3]	2 [0,4]	2 [0,3]	0	

^a^ Signifikante Unterschiede zwischen allen Kursen, *p* < 0,05

^b^ Signifikante Unterschiede zwischen Hämatologiekurs und den jeweils anderen Kursen

^c^ Mehrfachnennungen möglich

^d^ Zahlen geschätzt nach C. Frerk [29]

## Ergebnisse

Im Umfragezeitraum fanden 7 Kurse mit insgesamt 1417 Kursteilnehmerinnen und Kursteilnehmern statt. Von diesen wurden 1165 eingeschlossen. Nach Ausschluss von Datensätzen mit nicht plausiblen Zeitstempeln resultierte mit 1163 gültigen Datensätzen eine Rücklaufquote von 82,1 %.

### Soziodemografische Merkmale.

Die Befragten waren zu 93,3 % Ärztinnen und Ärzte in Weiterbildung. 88,1 % der Teilnehmerinnen und Teilnehmer hatten maximal 5 Jahre Berufserfahrung. 53,7 % waren christlich, 32,4 % konfessionsfrei und 11,9 % muslimisch. Weitere demografische Angaben sind in Tab. 2 dargestellt.

### Kenntnisse zum Urteil des Bundesverfassungsgerichts (BVerfG) und zu Gesetzesvorschlägen.

**Figure 1:**
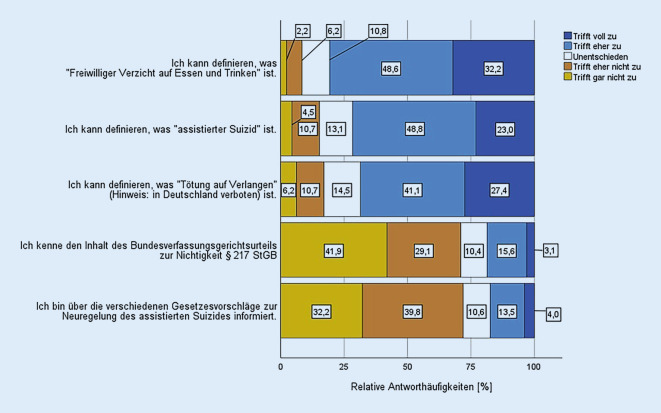

71,0 % der Befragten kannten den Inhalt des BVerfG-Urteils zum § 217 nicht. 72,0 % waren über die Gesetzesvorschläge zur Neuregelung des assistierten Suizides nicht informiert (Abb. 1).

### Vorerfahrungen.

**Figure 2:**
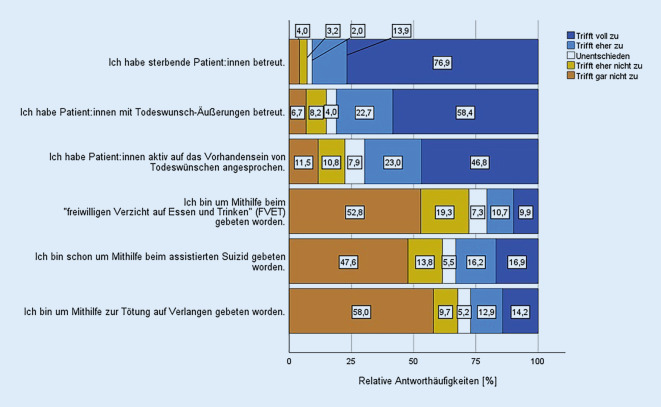

Zum Zeitpunkt der Untersuchung hatten 90,8 % der Teilnehmerinnen und Teilnehmer bereits sterbende Patientinnen und Patienten betreut (Abb. 2). 33,1 % wurden bereits um eine Suizidassistenz gebeten, 27,1 % um eine Tötung auf Verlangen, 20,7 % wurden um Unterstützung beim freiwilligen Verzicht auf Essen und Trinken (FVET) gebeten. 38 Befragte (3,3 %) hatten schon persönlich bei einer Suizidassistenz mitgewirkt (nicht dargestellt).

### Einstellungen und Haltungen.

**Figure 3:**
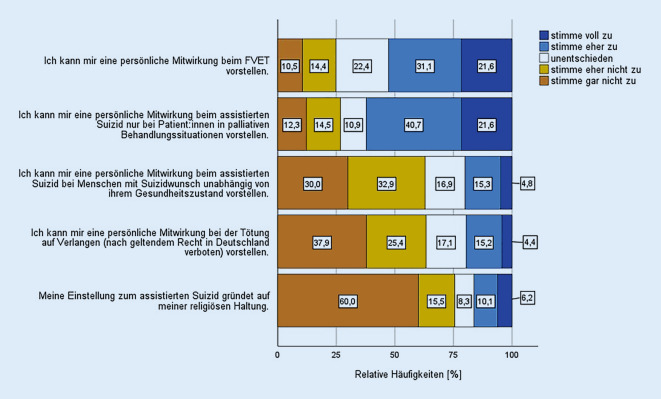

62,3 % konnten sich eine persönliche Mitwirkung an einem assistierten Suizid nur in palliativen Behandlungssituationen vorstellen, 10,9 % der Befragten waren in dieser Frage unentschieden (Abb. 3). Demgegenüber konnten sich 20,1 % eine persönliche Mitwirkung bei einer Suizidassistenz bei Menschen mit Suizidwunsch unabhängig von deren Gesundheitszustand vorstellen, was 62,9 % ablehnten und 16,9 % unentschieden beantworteten. Ähnlich wurde die Frage nach einer persönlichen Mitwirkung bei einer – in Deutschland verbotenen – Tötung auf Verlangen beantwortet, die sich 19,6 % der Befragten vorstellen konnten, während 63,3 % sie ablehnten, 17,1 % äußerten sich unentschieden. 52,7 % konnten sich eine persönliche Mitwirkung beim FVET vorstellen, 24,9 % lehnten diese ab und 22,4 % äußerten sich hierzu unentschieden. Keine Ärztin und kein Arzt mit der Zusatzbezeichnung Palliativmedizin lehnte eine persönliche Mitwirkung beim FVET ab. Bei 16,3 % der Teilnehmerinnen und Teilnehmer gründete die Einstellung zum assistierten Suizid auf ihrer religiösen Einstellung.

### Einflussfaktoren auf die Einstellungen zur Suizidassistenz bei Menschen mit Suizidwunsch unabhängig von ihrem Gesundheitszustand

Die Einstellung der Teilnehmerinnen und Teilnehmer zur Suizidassistenz bei Menschen mit Suizidwunsch, unabhängig von deren Gesundheitszustand, erwies sich als unabhängig vom belegten Kurs, der Fachrichtung, der Zahl der Berufsjahre, den erworbenen Zusatzbezeichnungen, dem Facharztstatus, dem Geschlecht, den Vorerfahrungen mit Sterbenden oder Todeswünschen sowie den inhaltlichen Kenntnissen des Bundesverfassungsgerichtsurteils zum § 217.

Befragte, die über das aktuelle Gesetzgebungsverfahren informiert waren, konnten sich eine Teilnahme an einer Suizidassistenz bei Menschen mit Suizidwunsch unabhängig von deren Gesundheitszustand häufiger vorstellen (41,3 % befürwortend, 52,2 % ablehnend, r = 0,180, *p* < 0,05). Das Gleiche traf auf Teilnehmende zu, die sich bereits eine klare Einstellung zum assistierten Suizid erarbeitet hatten (r = 0,180, *p* > 0,001).

Von den befragten Ärztinnen und Ärzten muslimischen Glaubens lehnten 76,4 % eine Teilnahme an der Suizidassistenz bei Menschen mit Suizidwunsch unabhängig von deren Gesundheitszustand ab (demgegenüber 14,2 % zustimmend), bei den Christen waren es 66,6 % (17,2 % zustimmend), bei den Konfessionsfreien 54,9 % (25,3 % zustimmend; paarweise U‑Tests, *p* < 0,001, r = −0,127/0,161/−0,273 bei Christentum vs. konfessionsfrei/Christentum vs. Islam/Islam vs. konfessionsfrei).

Die Befragten, die angaben, dass ihre Haltung zur Suizidassistenz auf ihrer Religion gründete, äußerten häufiger eine ablehnende Haltung zur Teilnahme an einer Suizidassistenz bei Menschen mit Suizidwunsch unabhängig von deren Gesundheitszustand (r = −0,153, *p* < 0,001). Die Haltungen von Teilnehmerinnen und Teilnehmern, die einen Einfluss ihrer religiösen Einstellung verneinten – egal ob christlich, islamisch oder konfessionsfrei –, unterschieden sich nicht (*p* = 0,9).

Die Einstellung zum assistierten Suizid bei Menschen mit Suizidwunsch unabhängig von deren Gesundheitszustand korrelierte positiv mit der Einschätzung, dass die Suizidbeihilfe eine ärztliche Aufgabe sei (*p* < 0,001, r = 0,178), und negativ mit der Einschätzung, dass hierdurch das Vertrauen in die Ärzteschaft beschädigt werde (*p* < 0,001, r = −0,224).

Diejenigen, die bereits um eine Suizidassistenz gebeten wurden, konnten sich eine Teilnahme an einem assistierten Suizid bei Menschen mit Suizidwunsch unabhängig von deren Gesundheitszustand häufiger vorstellen als solche, die bisher nicht darum gebeten wurden (*p* < 0,001, r = 0,164).

### Einflussfaktoren auf die Einstellungen zur Suizidassistenz in palliativen Behandlungssituationen

Die Einstellung zur Suizidassistenz bei Patientinnen und Patienten in palliativen Behandlungssituationen erwies sich als unabhängig vom belegten Kurs, der Fachrichtung, der Zahl der Berufsjahre, den erworbenen Zusatzbezeichnungen, dem Facharztstatus, dem Geschlecht, den Vorerfahrungen mit Sterbenden oder Todeswünschen, der Frage, ob die Befragten sich bereits eine klare Einstellung zum assistierten Suizid erarbeitet hatten, sowie den Kenntnissen über das Bundesverfassungsgerichtsurteil zum § 217 oder das aktuelle Gesetzgebungsverfahren.

Von den teilnehmenden Ärztinnen und Ärzten muslimischen Glaubens lehnten 50,4 % eine Teilnahme an der Suizidassistenz nur bei Menschen in palliativen Behandlungssituationen ab (41,0 % zustimmend, 8,7 % unentschieden, *p* < 0,001, r = −0,258). Von den nicht muslimischen Teilnehmenden antworteten 24 % ablehnend, 11,2 % neutral und 64,9 % zustimmend. Die Einstellungen zur Suizidassistenz in palliativen Behandlungssituationen von Teilnehmerinnen oder Teilnehmern anderer Konfessionen unterschieden sich nicht.

Die Befragten, die angaben, dass ihre Haltung zur Suizidassistenz auf ihrer Religion gründete, äußerten zur Teilnahme an einer Suizidassistenz in palliativen Behandlungssituationen häufiger eine ablehnende Haltung (r = −0,234, *p* < 0,001).

Die Einstellung zum assistierten Suizid bei Menschen mit Suizidassistenz nur in palliativen Behandlungssituationen korrelierte positiv mit der Einschätzung, dass die Suizidbeihilfe eine ärztliche Aufgabe sei (*p* < 0,001, r = 0,303), und negativ mit der Einschätzung, dass hierdurch das Vertrauen in die Ärzteschaft beschädigt werde (*p* < 0,001, r = −0,277).

### Vorstellungen zu normativen Regelungen

**Figure 4:**
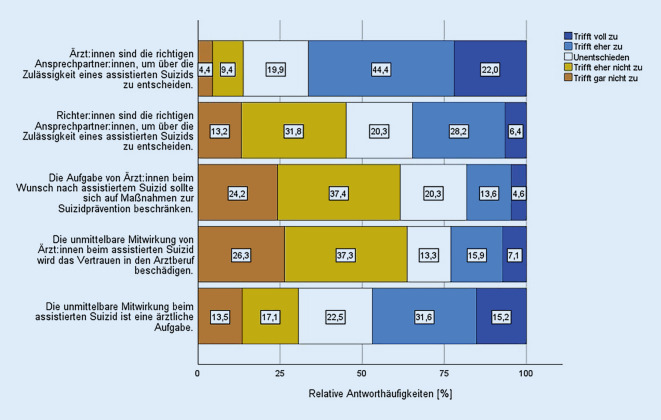

66,4 % befürworteten die Aussage, dass Ärztinnen und Ärzte die richtigen Ansprechpartner sind, um über die Zulässigkeit eines assistierten Suizids zu entscheiden (Abb. 4). 19,9 % waren unentschieden und für 13,8 % waren Ärztinnen und Ärzte diesbezüglich nicht die richtigen Ansprechpartner. Für 34,6 % waren Richterinnen und Richter die richtigen Ansprechpartner, um über die Zulässigkeit eines assistierten Suizids zu entscheiden. 45,0 % stimmten dem nicht zu und 20,3 % waren hierzu unentschieden.

Eine Mehrheit von 61,6 % der Befragten lehnte die Aussage ab, dass Ärztinnen und Ärzte sich bei einem Wunsch nach assistiertem Suizid auf Maßnahmen zur Suizidprävention beschränken sollten.

23,0 % der Befragten sahen durch die unmittelbare Mitwirkung von Ärztinnen und Ärzten beim assistierten Suizid das Vertrauen in den Arztberuf beschädigt. 63,6 % teilten diese Befürchtung nicht, 13,3 % waren in dieser Frage unentschieden.

46,8 % stimmten der Aussage zu, dass die Mitwirkung beim assistierten Suizid eine ärztliche Aufgabe sei, 30,6 % stimmten nicht zu und 22,5 % waren in dieser Frage unentschieden.

## Diskussion

In der vorliegenden Umfrage wurden Daten von 1163 klinisch tätigen Ärztinnen und Ärzten, zumeist mit einer Berufserfahrung von weniger als 5 Jahren, ausgewertet. Damit beruht die vorliegende Studie nach unserem Kenntnisstand auf einer der größten bisher publizierten Stichproben bzgl. ärztlicher Suizidassistenz [15]. Sie ist zudem eine der ersten mit Fokus auf die junge Ärzteschaft. In unserer Umfrage zeigte sich, im Gegensatz zu früheren [14, 20], wenngleich nicht allen [21] Erhebungen, kein Zusammenhang zwischen der Berufserfahrung und der Einstellung zum ärztlich assistierten Suizid. Dies könnte auf der geringen Berufserfahrung der allermeisten Teilnehmenden beruhen, wodurch die Varianz der Einstellungen gering war.

Fast alle Teilnehmenden hatten bereits Kontakt zu sterbenden Patientinnen oder Patienten, was die Notwendigkeit der Etablierung von Unterstützungsangeboten zur Bewältigung dieser herausfordernden Aufgabe unterstreicht [22].

20,1 % unserer Stichprobe befürworteten eine Suizidassistenz bei Menschen mit Suizidwunsch unabhängig von deren Gesundheitszustand. Demgegenüber befürworteten mit 62,3 % mehr als 3‑mal so viele Teilnehmende dieser Studie eine persönliche Mitwirkung an einer Suizidassistenz nur in palliativen Behandlungssituationen. Damit machen Teile der Befragten ihre Entscheidung, bei einer Suizidassistenz mitzuwirken, offensichtlich im Rahmen einer individuellen Gewissensentscheidung vom Vorhandensein einer lebenslimitierenden Erkrankung abhängig. Die Zustimmungsraten waren damit höher als in Kollektiven von Psychiaterinnen und Psychiatern, multiprofessionellen Palliativexpertinnen und -experten oder Onkologinnen und Onkologen [17, 18, 23].

Übereinstimmend mit internationalen Studien korrelierten nur die demografischen Merkmale Religion und Religiosität mit der Haltung zum assistierten Suizid [15, 24]. Allerdings stellte Religiosität („Meine Einstellung zum assistierten Suizid gründet auf meiner religiösen Einstellung“) nur für 16,3 % in unserer Stichprobe einen relevanten Faktor für ihre Haltung zum assistierten Suizid dar, was die Frage aufwirft, welchen Stellenwert die Vorstellungen der Religionsgemeinschaften auf mögliche Regelungen haben sollten [25, 26].

In unserer Studie gaben 38 Personen (3,3 %) an, bereits bei einer Suizidassistenz persönlich mitgewirkt zu haben. Dies entspricht bekannten Dimensionen, wenngleich es überrascht, dass sie auch bei jungen Ärztinnen und Ärzten bereits eine solche Größenordnung hat [27].

Bemerkenswert ist, dass die Teilnehmenden in unserer Studie häufiger um Suizidassistenz als um Begleitung beim freiwilligen Verzicht auf Essen und Trinken (FVET) gebeten wurden. Ebenso können sich mehr Befragte eine Mitwirkung an einer Suizidassistenz in palliativen Behandlungssituationen als an einem FVET vorstellen. Möglicherweise ist die Bandbreite der palliativen Interventionen und Suizidalternativen sowohl Patientinnen und Patienten als auch den Teilnehmerinnen und Teilnehmern in unserer Befragung unzureichend bekannt. Dies würde die Position untermauern, dass weiterhin Aufklärungsarbeit über die Möglichkeiten der Palliativversorgung geleistet werden und eine frühzeitigere Integration dieser Versorgung erfolgen muss [6]. Wie auch in früheren Untersuchungen beobachtet, wurde der FVET von keinem der Befragten mit Zusatzbezeichnung „Palliativmedizin“ abgelehnt [28].

Unter den Teilnehmerinnen und Teilnehmern herrschte, anders als in Stellungnahmen von Fachgesellschaften und Fachgremien [6–8], Uneinigkeit in der Frage, ob Suizidassistenz eine ärztliche Aufgabe sei. Eine Mehrheit der von uns Befragten aber sah Ärztinnen und Ärzte als die richtigen Ansprechpartner, um über die Zulässigkeit eines assistierten Suizids zu entscheiden. Ebenso wollte eine Mehrheit die ärztlichen Aufgaben bei suizidwilligen Personen nicht auf Maßnahmen zur Suizidprävention beschränkt sehen.

Das Argument, dass die unmittelbare Mitwirkung beim assistierten Suizid das Vertrauen in die Ärzteschaft beschädige, teilte weniger als ein Viertel der Befragten. Eine deutliche Mehrheit widersprach damit in diesem Punkt der Position der Bundesärztekammer [8]. Nur etwas weniger als die Hälfte aller Befragten teilt die Position der Deutschen Gesellschaft für Psychiatrie und Psychotherapie, Psychosomatik und Nervenheilkunde, dass Gerichte die richtigen Ansprechpartner sind, um über die Zulässigkeit einer Suizidassistenz zu entscheiden [7].

Ein nach der medialen Berichterstattung über das Urteil des Bundesverfassungsgerichts vom Februar 2020 überraschendes Ergebnis war die häufig angegebene Unkenntnis über den genauen Inhalt des Urteils. 41,9 % der Befragten waren die Inhalte des Urteils gar nicht bekannt, nur 18,7 % gaben inhaltliche Kenntnisse an. Ähnlich gering waren mit 17,5 % die Teilnehmerinnen und Teilnehmer mit Kenntnis der bislang vorliegenden und nun vom Bundestag abgelehnten Gesetzentwürfe. Einerseits scheinen die Auseinandersetzung und Meinungsbildung, gemessen an der geringen Anzahl der als „unentschieden“ geäußerten Haltungen, auch unter jüngeren Ärztinnen und Ärzten weit fortgeschritten zu sein. Andererseits scheint es an einer ausreichenden Auseinandersetzung mit den geltenden normativen Rahmenbedingungen zu mangeln, sodass hier dringend weitere Aufklärungsarbeit notwendig ist. Denkbar wäre auch, dass jüngere Ärztinnen und Ärzte sich wenig in die Erarbeitung sowohl von Empfehlungen ärztlicher Gremien als auch von normativen Regelungen eingebunden fühlen. Ein interessantes Ergebnis scheint in diesem Zusammenhang, dass ein genauerer Kenntnisstand über Entscheidungen und Entwürfe in unserer Befragung mit einer höheren Zustimmung zum ärztlich assistierten Suizid einherging.

### Stärken und Limitationen

Die Stärke dieser Umfrage ist die Fokussierung auf die Haltungen jüngerer Ärztinnen und Ärzte, die in der bisherigen Debatte bislang wenig Raum einnahm. Des Weiteren konnten wir im Vergleich zu bisherigen Erhebungen an einer großen Stichprobe eine hohe Rücklaufquote und reliable Ergebnisse erzielen. Ein häufiges Problem derartiger Studien ist die niedrige Validität der Fragebögen [15]. So bleibt auch die Auswahl unserer Fragen ohne externe Validierung. Dass die genaue Formulierung der gestellten Fragen eine große Rolle spielen kann, ist uns beispielsweise an: „Ich kann ich mir eine persönliche Mitwirkung beim FVET vorstellen“, deutlich geworden. So bleibt es der Interpretation der Befragten überlassen, ob eine persönliche Mitwirkung bereits mit der Betreuung entsprechender Patientinnen und Patienten beginnt oder erst bei der Verschreibung von Medikamenten.

Wir haben im Sinne der Zugänglichkeit des Fragebogens eine weitgehend konsequente Verwendung der 5‑stufigen Skala vom Likert-Typ angestrebt. Einzelne Fragen boten hierdurch fehlinterpretierbare Antwortmöglichkeiten (Beispiel: „Ich habe sterbende Patient:innen betreut“ – „Stimme eher zu“ vs. „Stimme voll zu“). Es ist möglich, dass Teilnehmerinnen oder Teilnehmer sich bei einer rein bipolaren Skala (Ja/Nein) anders geäußert hätten. Gerade bei bedingten Fragen („Ich kann mir eine persönliche Mitwirkung beim assistierten Suizid nur bei Patientinnen und Patienten in palliativen Behandlungssituationen vorstellen“) könnte eine negative Antwort sowohl auf eine restriktivere als auch eine weniger restriktive Haltung hinweisen. Beide Risiken werden durch die Tatsache gemildert, dass wir den Ordinalcharakter der Skala zusätzlich grafisch herausgestellt haben.

Mehrere Nachfolgeprojekte, die auf unserem Fragebogen aufbauen und andere Kollektive adressieren, befinden sich derzeit in unterschiedlichen Umsetzungsphasen. Auch der Ansatz, über die Kurse der Arbeitsgemeinschaft Intensivmedizin e. V. in Arnsberg Stichproben aus dem Kollektiv der „jungen Ärzteschaft“ zu rekrutieren, wird in mehreren Nachfolgeprojekten zu palliativmedizinischen und notfallmedizinischen Forschungsthemen weiterverfolgt. Unsere Studie kann in beiden Aspekten als Pilotprojekt verstanden werden.

Während die Zugehörigkeit zu christlichen Religionen der Verteilung der Gesamtbevölkerung entsprach, gab es in der Stichprobe im Vergleich zur Gesamtbevölkerung ein häufigeres Bekenntnis zum Islam. Da die Zugehörigkeit zum Islam negativ mit der Bereitschaft zur Teilnahme an einem assistierten Suizid korrelierte, kann hierdurch eine Verzerrung der Ergebnisse im Sinne einer Unterschätzung der Teilnahmebereitschaft an Suizidassistenz, FVET und Tötung auf Verlangen vorliegen.

## Fazit

Die Untersuchung zeigt, dass jüngere Kolleginnen und Kollegen eine eher liberale Haltung zur Suizidassistenz aufweisen. In unserer Stichprobe differenzierten die Befragten in ihrer Haltung zur Suizidassistenz stark zwischen Menschen ohne Vorerkrankungen und Patientinnen und Patienten in palliativen Behandlungssituationen. Überwiegend wurden Ärztinnen und Ärzte, und nicht Richterinnen und Richter, als die richtigen Ansprechpartner bei der Frage nach der Zulässigkeit eines Suizidwunsches gesehen. Weitere Untersuchungen über die Ursachen der häufigen Unkenntnis der normativen Grundlagen sind notwendig. Die Ergebnisse legen nahe, dass mehr Aufklärungsarbeit über Suizidalternativen und palliativmedizinische Versorgungsmöglichkeiten geleistet werden muss.
